# Neuroligin-1 Overexpression in Newborn Granule Cells *In Vivo*

**DOI:** 10.1371/journal.pone.0048045

**Published:** 2012-10-22

**Authors:** Eric Schnell, AeSoon L. Bensen, Eric K. Washburn, Gary L. Westbrook

**Affiliations:** 1 Portland VA Medical Center, Portland, Oregon, United States of America; 2 OHSU Department of Anesthesiology and Perioperative Medicine, Portland, Oregon, United States of America; 3 The Vollum Institute, Oregon Health & Science University, Portland, Oregon, United States of America; University of South Florida, United States of America

## Abstract

Adult-born dentate granule cells integrate into the hippocampal network, extend neurites and form synapses in otherwise mature tissue. Excitatory and inhibitory inputs innervate these new granule cells in a stereotyped, temporally segregated manner, which presents a unique opportunity to study synapse development in the adult brain. To examine the role of neuroligins as synapse-inducing molecules in vivo, we infected dividing neural precursors in adult mice with a retroviral construct that increased neuroligin-1 levels during granule cell differentiation. By 21 days post-mitosis, exogenous neuroligin-1 was expressed at the tips of dendritic spines and increased the number of dendritic spines. Neuroligin-1-overexpressing cells showed a selective increase in functional excitatory synapses and connection multiplicity by single afferent fibers, as well as an increase in the synaptic AMPA/NMDA receptor ratio. In contrast to its synapse-inducing ability in vitro, neuroligin-1 overexpression did not induce precocious synapse formation in adult-born neurons. However, the dendrites of neuroligin-1-overexpressing cells did have more thin protrusions during an early period of dendritic outgrowth, suggesting enhanced filopodium formation or stabilization. Our results indicate that neuroligin-1 expression selectively increases the degree, but not the onset, of excitatory synapse formation in adult-born neurons.

## Introduction

Adult-generated dentate granule cells have been implicated in learning [1], [2], [3], [4], [5], and dysregulation of neurogenesis has been linked to depression [6], schizophrenia [7], and epilepsy [8]. In animal models, such diseases can disrupt the rate of neurogenesis as well as synapse formation and network integration of newborn neurons [9]. Alterations in synapse formation and in the balance of circuit excitation and inhibition have been increasingly recognized in neurobehavioral disorders [10], [11], suggesting that appropriate integration of neurons is crucial to proper network function. The generation of newborn granule cells in the adult hippocampus provides an interesting model system in this regard, because these cells follow a stereotyped and temporally segregated pattern of synapse formation. As the dendrites of new granule cells increase in complexity and length, GABAergic inputs (weeks 1–2) precede excitatory innervation and spine formation (weeks 3–4) [12], [13], [14]. Eventually, these cells become functionally similar to granule cells generated much earlier in development [15], [16].

Molecular candidates for synapse formation in adult-born neurons have largely been inferred from studies during embryonic development [17]. In particular, the neuroligin (NLG) family of proteins (NLG1-4) [18] is thought to play an important role in synapse formation during early development [19], [20]. However, there are apparent discrepancies between the roles of neuroligins between *in vitro* and *in vivo* studies, mostly deduced from studies of the neuroligin-1 isoform. *In vitro*, neuroligin expression in non-neuronal cells is sufficient to induce functional synaptic connectivity with co-cultured neurons [21], [22], [23], suggesting an instructive role in synapse initiation and assembly. The neuroligin triple knockout mouse has a profound functional synaptic deficit and neonatal mortality [24], but synapse number and morphology are unperturbed, suggesting that neuroligins *in vivo* act at a stage subsequent to initial synapse formation. Likewise, neuroligin-1 overexpression *in vitro* can increase both excitatory and inhibitory synapses [25], [26], [27], [28], whereas *in vivo* studies have suggested that neuroligin-1 is selective for excitatory synapses [29], [30], [31].

We took advantage of the temporally segregated onset of glutamatergic and GABAergic synapses in adult-generated newborn granule cells to examine the synapse specificity of neuroligin-1 function at different stages of differentiation. Using viral-mediated gene transfer *in vivo*, we increased neuroligin-1 levels in newborn neurons in the hippocampus of young adult mice. Retroviral infection prior to the onset of synapse formation caused a selective increase in excitatory synapses as well as an increase in filopodial-like dendritic protrusions, but did not alter the timing of synaptogenesis nor inhibitory synaptic function.

## Materials and Methods

### Ethics statement

All viral injections were carried out under isoflurane anesthesia followed by oral post-procedural acetaminophen for 2 days to minimize animal discomfort using OHSU/Portland VA Institutional Animal Care and Use Committee and Biosafety Committee approved protocols (OHSU IACUC protocol IS00000455, PVAMC IACUC protocol 2679) in accordance with NIH guidelines for the ethical treatment of animals. Mice were deeply anesthetized and decapitated prior to brain tissue removal.

### Preparation of viral vectors

**Figure 1:**
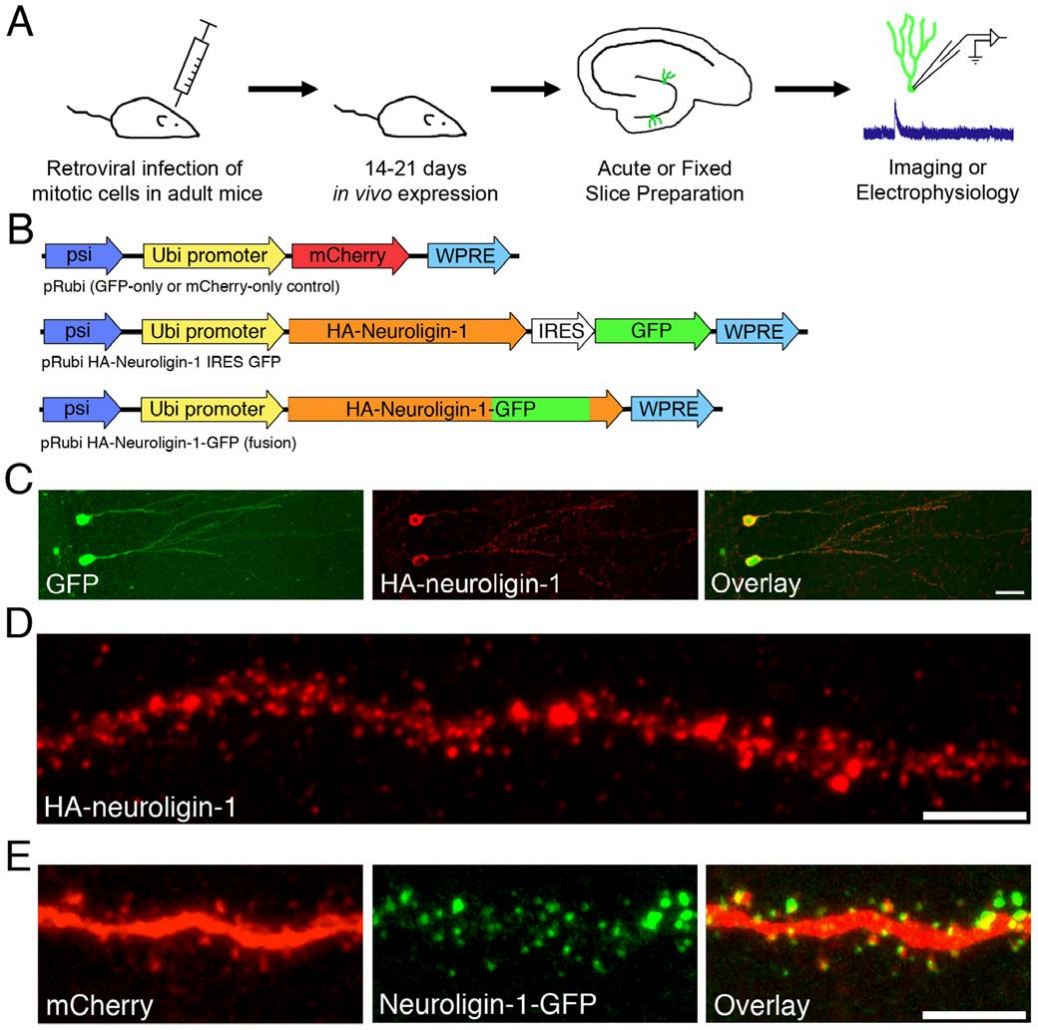
Retrovirus-mediated neuroligin-1 expression in newborn granule cells. A. Experimental design schematic illustrating retroviral infection of dentate stem cells in anesthetized mice, allowing *in vivo* cell maturation, and subsequent *ex vivo* imaging or recording at defined post-mitotic stages (14 or 21 dpi). B. Retroviral constructs used in this paper. The Ubiquitin promoter drives expression of control proteins (either GFP or mCherry), a neuroligin-1-GFP fusion protein, or neuroligin-1 in conjunction with GFP via an IRES sequence. All neuroligin-1 constructs also carried an extracellular HA tag. C. Newly born granule cells 21 days post-mitosis, infected with HA-neuroligin-1 IRES GFP retrovirus at day 0. Confocal stacks of anti-HA and anti-GFP stained granule cells demonstrate co-expression of exogenous neuroligin-1 and GFP. Scale bar: 20 μm. D. Higher power image of an infected granule cell dendrite stained with anti-HA antibody (red), showing the exogenous HA-neuroligin-1 expression pattern. Scale bar: 5 μm. E. High-power image of a dendritic segment from a double-infected granule cell (one retrovirus encoding mCherry to outline cell morphology, and a separate virus encoding a neuroligin-1-GFP fusion protein). Scale bar, 5 μm.

Moloney Murine Leukemia Virus (MMLV)-based retroviral vectors require cell mitosis to achieve expression [32], and thus can specifically target newly-born granule cells [15]. We created pseudotyped, replication-deficient MMLV particles using established protocols [33] with a pSie-based viral genomic backbone [34]. Briefly, the MMLV 5' and 3' long terminal repeat regions and Psi packaging sequences were used to flank hemaglutinin (HA)-tagged mouse neuroligin-1 containing both the A and B alternatively-spliced inserts (courtesy of Anne-Marie Craig) [35], under control of a ubiquitin promoter. Green Fluorescent Protein (GFP) was co-expressed under the control of an internal ribosome entry site (IRES) sequence (Figure 1A, B). Retroviruses encoding only GFP or mCherry were used to infect cells in separate age-matched mice, and to provide time-matched control data for physiology and imaging experiments. For some morphology experiments, an HA-neuroligin-1-GFP fusion protein was created by placing GFP in frame in the C-terminal tail of HA-neuroligin-1 at its RsrII site, and sub-cloned into our retroviral vector using standard techniques.

### Intrahippocampal injection and tissue preparation

1-10×10^6^ viral particles were stereotaxically injected into the dorsal dentate gyri of 6–8 week old mice under isoflurane anesthesia. Mice recovered for 2–3 weeks prior to use in morphology and physiology experiments. At the designated post-injection interval, mice were terminally anesthetized, and transcardially perfused with choline chloride-based solution for acute hippocampal slices (see below) or fixative (3.7% paraformaldehyde with 4% sucrose in phosphate-buffered saline (PBS)) for morphology experiments.

### Electrophysiology

Hippocampi were sectioned (Leica VT1200S, 300 µM) in ice-cold solution containing (in mM): 110 CholineCl, 7 MgCl_2,_ 2.5 KCl, 1.25 NaH_2_PO_4*_2H_2_O, 0.5 CaCl_2_, 1.3 Na-ascorbate, 25 NaHCO_3_ bubbled with 95% O_2_-5% CO_2._ Live infected granule cells were identified in acute slices *in vitro* by combining fluorescence microscopy with infrared differential interference contrast imaging on a Zeiss Axioskop 2FS. Whole cell voltage-clamp recordings were made from infected cells using 3–5 MΩ glass micropipettes and an Axopatch 200B amplifier (Axon Instruments). Series resistance (R_s_) was monitored on-line, and cells were discarded if R_s_ changed by greater than 20%. Cesium gluconate-based pipette internal solution included (in mM): 100 gluconic acid, 10 EGTA, 10 HEPES, 17.5 CsCl, 8 NaCl, 2 Mg-ATP, 0.3 Na-GTP, pH = 7.3 (using 50% CsOH), 290 mOsm. Bath external solutions contained (in mM): 125 NaCl, 25 NaHCO_3_, 2.5 KCl, 1.25 NaH_2_PO_4_, 2.0 CaCl_2_, 1.0 MgCl_2_, and 25 D-glucose, bubbled with 95% O_2_-5% CO_2_. Cell input resistance and capacitance were measured from current transients in response to a hyperpolarizing voltage step. Excitatory postsynaptic currents (EPSCs) were recorded in the presence of 10 µM SR95531 to block GABA_A_ receptors. Inhibitory synaptic currents were recorded using a CsCl-based internal solution (in mM: 125 CsCl, 5 HEPES, 10 Cs-BAPTA, 2 Mg-ATP, 0.3 Li-GTP, neurobiotin 0.5%, pH = 7.2, 290 mOsm) while recording at -70 mV in the presence of 10 µM NBQX. Miniature post-synaptic currents were recorded in the presence of 1 µM tetrodotoxin (TTX). Miniature and spontaneous (without TTX) event amplitudes and frequencies were analyzed from a minimum of 5 minutes of continuous recording using an automated template-matching algorithm (Axograph). A bipolar stimulating electrode placed in the dentate middle molecular layer was used to evoke responses using an IsoFlex constant current stimulus isolation unit (A.M.P.I). The relative receptor composition at excitatory synapses was determined by evoking EPSCs at both −70 mV and +40 mV with the same stimulus intensity in a single cell, thus revealing the α-amino-3-hydroxyl-5-methyl-4-isoxazole-propionic acid receptor (AMPAR) and combined AMPAR- and N-methyl-D-aspartic acid receptor (NMDAR)-mediated currents, respectively. Relative NMDAR-only current amplitudes were estimated by measuring the evoked current amplitude at +40mV after the AMPAR-mediated component had completely decayed (60 ms latency). The decay phases of NMDAR-mediated currents (60–1000 ms after the stimulation) were fitted with double exponential curves. To assess the probability of presynaptic release, we measured the amplitude ratio of two AMPAR-mediated responses evoked at short intervals (50–250 ms). Groups were compared using two-tailed, unpaired t-tests, with n as the number of cells in each group.

### Immunohistochemistry and cell labeling

Brains were post-fixed overnight, washed x3 in PBS with 4% sucrose, and cut into 100–150 µm sections on a vibratome. Tissue sections were permeabilized with PBS containing 0.4% triton, blocked in PBS +10% horse serum, and stained using an anti-GFP Alexa 488 conjugated antibody (Invitrogen) and/or an anti-mCherry monoclonal antibody (Clontech) followed by an Alexa-568 conjugated secondary antibody. Anti-HA staining was performed with a monoclonal anti-HA antibody (clone 16B12, Covance). Apart from a subset of slices used to assess co-expression of GFP and exogenous neuroligin-1, all HA-stained sections were pre-treated with a citrate/heat-based antigen retrieval process that produced higher quality HA staining but a loss of GFP signal. Stained slices were mounted on glass slides using Fluoromount G mounting medium. In some cells, Lucifer Yellow CH (0.5%, Molecular Probes) or neurobiotin (0.5%, Vector) was added to the internal solution for dye-filling, and live, virus-infected cells were filled for >5 minutes at a membrane potential of -70 mV. These acute slices were then fixed for 30 minutes in paraformaldehyde- and sucrose-containing PBS, permeabilized and counterstained with streptavidin-DyLight549 (for neurobiotin fills only), washed, and mounted directly onto glass slides.

### Imaging

Stained slices were imaged using an inverted LSM-710 confocal microscope. For spine imaging, 2–3 separate 20–30 micron segments of dendrite from infected granule cells in the dorsal hippocampus were imaged in the middle molecular layer, using a 63x/1.4NA oil objective to acquire Z-stacks of the entire stretch. Images were manually quantified off-line using ImageJ software. For whole cell morphology, intact cells were imaged from 150 micron thick slices using a 40x/1.2NA objective, and manually traced using ImageJ. Cells were excluded if the staining was not bright enough to allow unambiguous identification of the distal extent of dendritic branches, or if the dendritic tree was clearly truncated by the plane of sectioning (for whole cell morphology). Sholl analyses were performed on traced cells using the Sholl Analysis plug-in (Ghosh Lab). All images were manipulated solely using linear transformations. Spine counts, dendritic lengths, and soma sizes were compared between groups using unpaired t-tests with n equal to the number of cells per group. Sholl analyses were compared at the respective radii using a two-tailed ANOVA for repeated measures, with a Bonferroni correction, using Prism software (GraphPad). All data are presented as mean ± standard error of the mean (SEM) unless otherwise noted.

## Results

### Exogenous neuroligin-1 targeted appropriately to the tips of dendritic spines in newborn neurons

Using intrahippocampal injections of a bicistronic retroviral vector in vivo, we overexpressed HA-tagged neuroligin-1 along with GFP in adult-generated newborn granule cells (Figure 1A–D). All GFP-positive cells were HA-immunoreactive, indicating faithful co-expression of both proteins in neurons that had the typical morphology of newborn granule cells. (Figure 1C). At 21 days post-injection, anti-HA staining demonstrated punctate localization of exogenous HA-neuroligin-1 (Figures 1C, D). Imaged at higher power, exogenous neuroligin-1 could be localized to the heads of dendritic spines (Figure 1D). Because the antigen retrieval process used to improve HA staining (Figure 1D) caused fading of GFP (data not shown), we also engineered a HA-neuroligin-1-GFP fusion construct by placing GFP in frame inside the C-terminal (intracellular) tail of HA-neuroligin-1. Anti-HA staining in non-antigen-retrieved slices demonstrated complete overlap between HA staining and GFP (data not shown). In mice co-infected with a retrovirus expressing mCherry as well as the HA-neuroligin-1-GFP fusion retrovirus, red fluorescence filled the cell, allowing visualization of dendritic shafts and spines (Figure 1E). The green fluorescence associated with the HA-neuroligin-1-GFP fusion protein was nearly completely localized to dendritic spines (Figure 1E), confirming appropriate synaptic targeting of exogenous neuroligin-1. Neuroligin-1-GFP was detectable in >90% of spines that were labeled with mCherry (n = 5 cells), suggesting that most, if not all, spines expressed exogenous neuroligin-1.

### Overexpression of neuroligin-1 increased dendritic spine number in adult-generated granule cells

**Figure 2:**
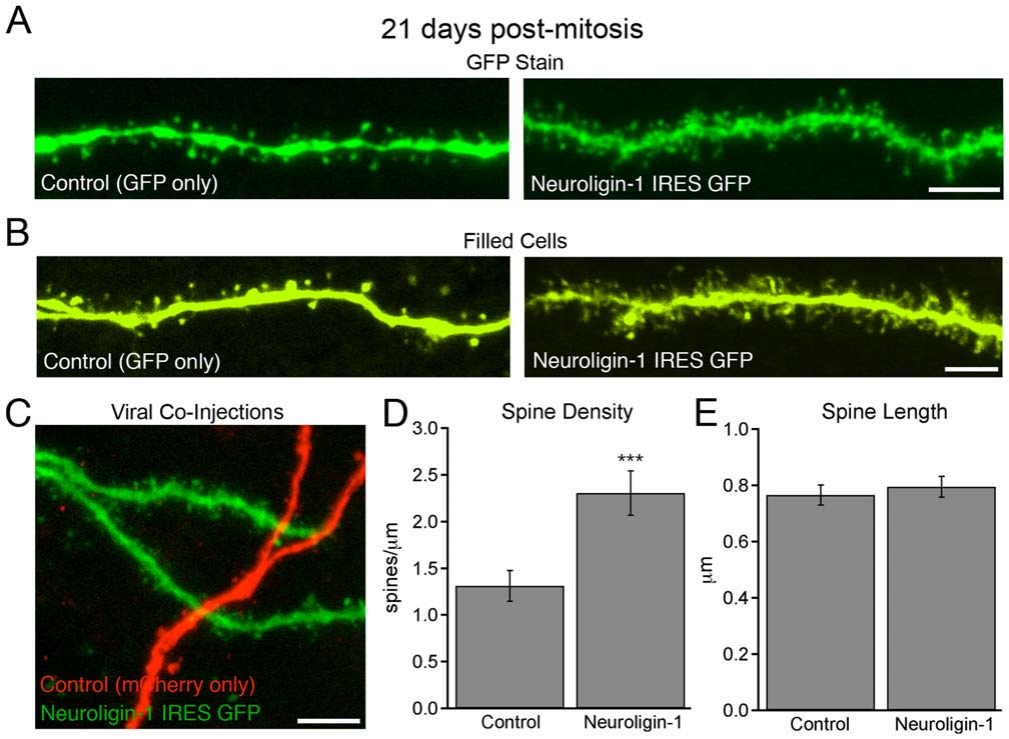
Neuroligin-1 overexpression increases dendritic spine number. A. Dendritic spine morphology as demonstrated from GFP expression in 21-day-old granule cells expressing GFP only (control) or HA-neuroligin-1 in conjunction with GFP. Scale bar: 5 μm. B. To control for variability between fluorophore expression levels, a subset of 21-day-old live cells was identified by GFP expression, filled with membrane-impermeant dye by patch pipette, and fixed. Images show sections of dendrite taken from 21-day-old control cells and neuroligin-1 overexpressing cells. Scale bar: 5 μm. C. The dentate middle molecular layer of an adult mouse co-injected with separate retroviruses expressing mCherry (control) or neuroligin-1 IRES GFP. The projected image shows a 21-day-old control granule cell (mCherry) next to a GFP-filled cell overexpressing neuroligin-1. Scale bar: 5 μm. D, E. Summary data of spine measurements from infected cells 21 days post-mitosis demonstrating an increase in spine density but no change in spine length. Control neurons were infected with a retrovirus expressing GFP only (data shown ± SEM; control, n = 11 cells; NLG-1, n = 10 cells; ***p<0.005).

In adult mice, newborn granule cells develop dendritic spines during their 3^rd^ post-mitotic week [36], which parallels their acquisition of functional excitatory synapses [12], [37]. Thus we first examined dendritic spine densities in newborn granule cells 21 days after infection with the HA-neuroligin-1 IRES GFP retrovirus. Even though IRES-driven GFP expression was generally lower than with the GFP-only control virus (unpublished observation, ES and ALB), neuroligin-1 overexpression almost doubled the spine density compared to GFP-only expressing control cells (control  = 1.31±0.17 spines/µm, n = 11 cells; NLG-1 = 2.30±0.24 spines/µm, n = 10 cells; p<0.005; Figure 2A, C, D). There was no change in spine length (control  = 0.77±0.04 µm, n = 11 cells; NLG-1 = 0.79±0.44 µm, n = 10 cells; p>0.6; Figure 2E).

To control for any effect of the reduced brightness of IRES-expressed GFP on spine counts, mCherry-only retroviruses were co-injected with the HA-neuroligin-1-GFP fusion construct. Using the red channel for quantification, we confirmed that spine density was greater in cells co-expressing neuroligin-1-GFP and mCherry (1.64±0.25 spines/µm, n = 5 cells) compared to those expressing mCherry alone (1.16±0.08 spines/µm, n = 10 cells, p<0.05). As the mCherry signal appeared slightly weaker in these cells than the GFP signal from GFP-only expressing cells, these numbers likely underestimate the absolute spine densities. In fact, spine densities in double-infected cells determined by counting the non-shaft neuroligin-1-GFP puncta in the green channel were higher (2.51±0.11 spines/µm), suggesting that some spines were not detectable in the red channel in these doubly-infected cells. The spine head width was marginally smaller in neuroligin-1 overexpressing cells (data not shown), indicating that an increase in spine size was not responsible for the increase in the number of dendritic spines. As a final test, we filled live, newborn granule cells by whole-cell patch pipette with a membrane-impermeant marker (Lucifer yellow or neurobiotin, Figure 2B), and subsequently fixed these slices for imaging. In these experiments, spine densities were lower than in perfusion-fixed brain tissue despite a very bright signal, suggesting that slice preparation or the dye-filling process itself caused destabilization or retraction of some spines. However, neuroligin-1 overexpressing cells still had higher spine densities compared to 21-day-old control (GFP-only) neurons (control  = 0.75±0.10 spines/µm, n = 9 cells; NLG-1 overexpression = 1.27±0.22 spines/µm, n = 6 cells, p<0.05). Thus using several different measurement strategies, neuroligin-1 overexpression led to a consistent increase in dendritic spine number.

### Neuroligin-1 selectively increased the number of functional excitatory synapses

**Figure 3:**
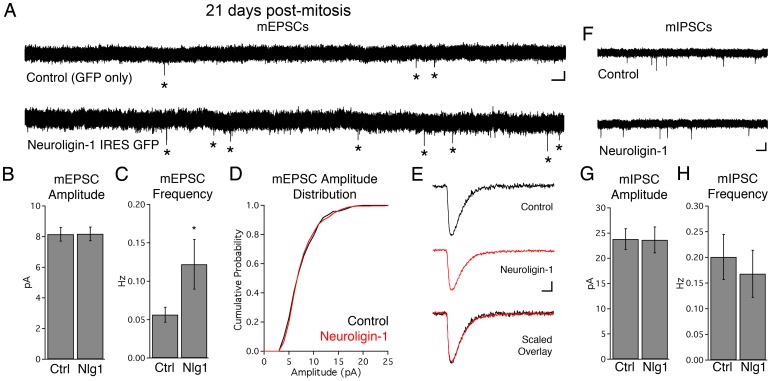
Neuroligin-1 overexpression increases the frequency of miniature excitatory post-synaptic currents in 3-week-old granule cells. A. Representative recordings showing mEPSCs in control (GFP only) and neuroligin-1 IRES GFP infected 21-day-old neurons. An asterisk is placed next to each detected event for clarity. Scale bars: 5 pA, 1 sec. B, C. Summary data for mEPSC amplitude and frequency (shown ± SEM; control n = 15 cells, NLG-1 n = 13 cells; * p<0.05). D. Cumulative mEPSC amplitude distribution for control (GFP only) and neuroligin-1 IRES GFP infected 21-day-old cells. E. Representative average mEPSCs are depicted for control (GFP only) and neuroligin-1 overexpressing cells. Scale bars for single traces: 2 pA, 5 ms. An overlay of peak-scaled responses demonstrates similar rise and decay kinetics between conditions. F, G, H. Neuroligin-1 overexpression does not change inhibitory innervation onto 21-day-old cells. F. Representative mIPSC recordings from control and neuroligin-1 overexpressing 21-day-old granule cells. Scale bars: 20 pA, 2 sec. G, H. Summary data for mIPSC amplitudes and frequencies at 21 dpi (shown ± SEM; control n = 14, NLG-1 n = 9; p>0.6 for each measurement).

We used whole cell voltage-clamp recordings from single infected newborn cells 21 days post-mitosis to examine the effect of neuroligin-1 overexpression on synaptic function. At this stage, newborn granule cells have begun acquiring excitatory glutamatergic inputs from the perforant path. Neuroligin-1 overexpression did not alter either whole cell capacitance or input resistance (control R_input_  = 998±141 MΩ, NLG-1 R_input_  = 1204±166 MΩ, p = 0.4; control C_m_  = 30.3±2.6 pF, NLG-1 C_m_  = 27.0±2.1 pF, p = 0.4; n = 15, 11 cells respectively). However, mEPSC frequency increased in neuroligin-1 overexpressing cells (control  = 0.06±0.01 Hz, n = 12 cells; NLG-1 = 0.12±0.03 Hz, n = 13 cells; p<0.05; Figure 3A, C) without a change in the distribution of mEPSC amplitudes (Figure 3B, D; p>0.7, Kolmogorov-Smirnov test), consistent with an increase in the number of active synapses without a change in the number of AMPA receptors per synapse. mEPSC rise times and decay kinetics were unaffected, suggesting that neuroligin-1 did not alter receptor kinetics or glutamate release dynamics (mEPSC 10-90% rise times: control  = 1.3±0.1 ms; NLG-1 = 1.3±0.1 ms; mEPSC half-width: control  = 5.8±0.4 ms; NLG-1 = 5.9±0.6 ms, n = 12, 13 cells respectively; p>0.7 each; Figure 3E).

Unlike overexpression of single neuroligin isoforms in cell cultures [25], [26], [27], [28], mIPSC amplitude and frequency were not altered by neuroligin-1 overexpression in newborn granule cells (Figure 3F–H). mIPSC kinetics also were unaltered by neuroligin-1 overexpression (mIPSC 10-90% rise time: control  = 2.1±0.2 ms, NLG-1 = 1.9±0.1 ms, p = 0.5; mIPSC half-width: control  = 17.2±1.2 ms, NLG-1 = 18.5±0.8 ms, p = 0.4; n = 14, 9 cells respectively). We obtained similar results for spontaneous IPSCs recorded in the absence of TTX (sIPSC amplitude: control  = 31.7±4.1 pA, NLG-1 = 30.4±2.8 pA, p = 0.8; sIPSC frequency: control  = 0.78±0.20 Hz, NLG-1 = 0.37±0.07 Hz, p>0.1, n = 13, 10 cells).

**Figure 4:**
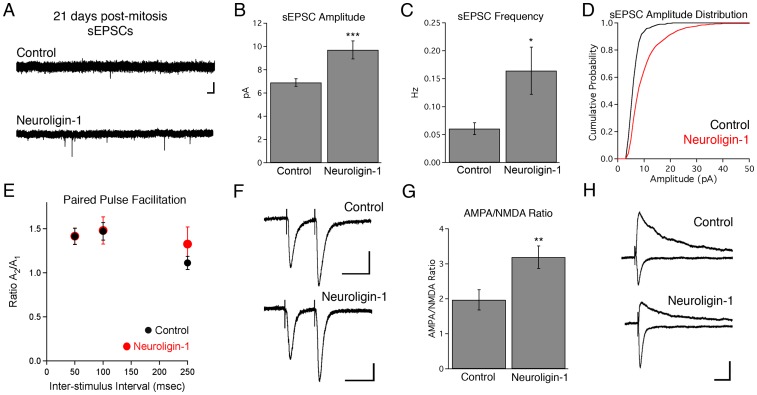
Neuroligin-1 overexpression increases the amplitude and frequency of spontaneous excitatory post-synaptic currents in 3-week-old granule cells. A. Representative sEPSC recordings from control (GFP only) and neuroligin-1 IRES GFP infected 21-day-old neurons in the absence of TTX. Scale bars: 10 pA, 500 ms. B, C. Summary data for sEPSC amplitude and frequency in 21-day-old adult-born neurons (n = 13 cells in each group, data shown ± SEM; * p<0.05, *** p<0.005). D. sEPSC amplitude distributions skew toward larger event amplitudes in neuroligin-1 overexpressing cells (p<0.0005, K-S test). E. Paired pulse facilitation is not altered in 21-day-old neuroligin-1 overexpressing cells relative to control (GFP only) cells. PPF is represented as the ratio of the peak amplitude of the second current to that of the first (control, n = 13,4,4 cells; NLG-1, n = 18,6,6 for 50, 100, and 250 ms intervals respectively; p>0.2 all groups). F. Representative traces at a 50 ms interval are shown for control and neuroligin-1 overexpressing 21-day-old neurons. Scale bars: 10 pA (Control), 20 pA (Neuroligin-1), 50 ms (both). G. Neuroligin-1 overexpression increases the ratio of AMPAR-mediated currents relative to NMDAR-mediated currents in 21-day-old granule cells (shown ± SEM; control, n = 16 cells; NLG-1, n = 12 cells; ** p<0.01). H. Sample recordings showing AMPAR-mediated currents (downward deflections) and slower combined AMPAR/NMDAR-mediated currents (upward deflections) recorded from single granule cells. Scale bars: 20 pA, 50 ms.

In the absence of TTX, newborn neurons overexpressing neuroligin-1 not only had an increase in spontaneous EPSC (sEPSC) frequency, but also an increase in sEPSC amplitude relative to control 21-day-old neurons (Figure 4A–C). The increase in sEPSC amplitudes resulted from a subset of significantly larger amplitude spontaneous events compared to age-matched control cells (Figure 4D; K-S test, p<0.0005). In control cells, the amplitude distribution of sEPSCs very closely matched that of mEPSCs, suggesting that normally there either were very few action potential-dependent events, or that action potentials only led to the release of single vesicles onto any particular postsynaptic cell. However, in neuroligin-1 overexpressing cells, the presence of significantly larger spontaneous events suggested that some action potentials now triggered simultaneous release of multiple glutamate quanta. Although postsynaptic neuroligin overexpression has been reported to increase the probability of release from presynaptic terminals [38], [39], neuroligin-1 overexpression in our experiments did not alter the paired pulse facilitation [40] of closely spaced evoked responses (50–250 ms, Figure 4E, F), indicating that the probability of neurotransmitter release onto infected cells was unchanged. Thus, the increased sEPSC amplitude likely reflects an increased number of connections between a neuroligin-1 overexpressing cell and an individual presynaptic axon.

Granule cells overexpressing neuroligin-1 also displayed an increased AMPAR/NMDAR current ratio (Figure 4G, H), as has been observed during synaptic maturation in the CA1 region of the hippocampus [41], [42]. As adult-born granule cells differentiate, there is also a shift in NMDAR subtype expression towards a faster-decaying isoform [43]. However, the NMDA current decay was well fit with a double exponential with similar kinetics in both groups (control %_fast_  = 95.7±0.9%; τ_fast_  = 79.0±10.6 ms, %_slow_  = 4.3±0.9%, τ_slow_  = 1410±633 ms, n = 14 cells; NLG-1 %_fast_  = 95.6±1.2%; τ_fast_  = 80.6±13.3 ms, %_slow_  = 4.4±1.2%, τ_slow_  = 649±124 ms, n = 11 cells; p>0.5 for all measurements).

### Neuroligin-1 overexpression did not accelerate the onset of excitatory innervation

**Figure 5:**
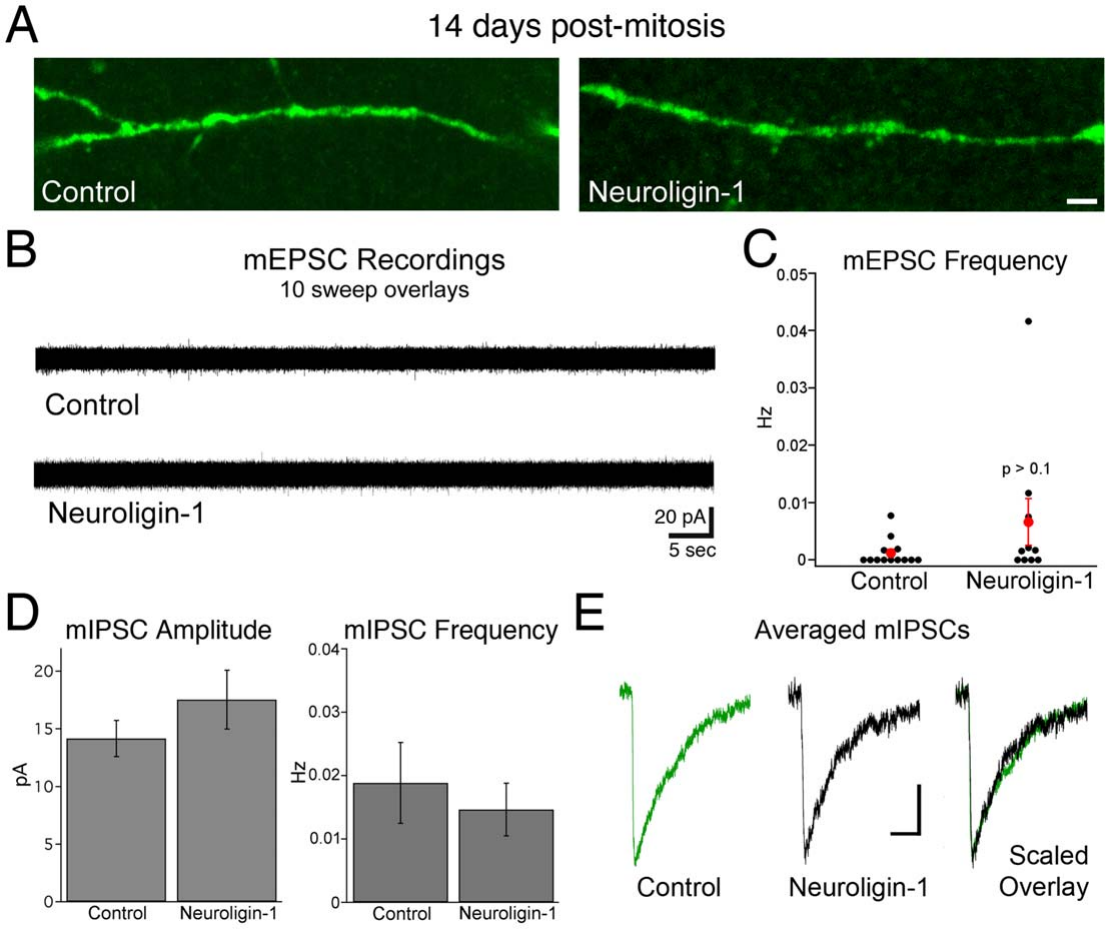
Neuroligin-1 overexpression does not accelerate excitatory synapse formation. A. Dendritic segments (inner molecular layer) of control and neuroligin-1 overexpressing granule cells rarely contain spines at 14 days post-mitosis. Scale bar: 2 µm. B. Sample mEPSC traces from control (GFP only infected) or neuroligin-1 overexpressing cells showing a lack of excitatory currents in 14-day-old cells. For each example, 10 consecutive sweeps (400 continuous seconds each) are overlaid. Scale bars: 20 pA, 5 sec. C. mEPSC frequencies from 14 day-old cells. The event frequency is shown for each cell, with mean data in red. The mode frequency in each group is zero events, and averaged frequencies are not significantly different (control, n = 13 cells; NLG-1, n = 10 cells, p>0.1). D. mIPSC recordings show no change in mIPSC amplitude or frequency in 14-day-old neuroligin-1 overexpressing cells (control, n = 10 cells; NLG-1, n = 8 cells; p>0.2 for each). E. Representative averaged mIPSCs from single cells, as well as a peak-scaled overlay, demonstrate no difference in mIPSC kinetics between conditions. Scale bars: 50 pA, 50 ms.

At 14 days post-mitosis, adult-generated granule cells normally have not yet acquired significant functional excitatory synaptic innervation. We used retroviruses to identify and label newborn control granule cells at this post-mitotic stage. Dendritic spines were rarely observed in these neurons, as was excitatory synaptic activity (9/13 cells with no mEPSCs, 8 mEPSCs in 90 minutes of recording from 13 cells, Figure 5A–C), consistent with prior observations [12], [37], [44]. As neuroligin-1 overexpression can induce synapse formation onto heterologous cells, we examined whether excitatory synapse formation could be accelerated in newborn neurons *in vivo* by increasing neuroligin-1 levels. Anti-HA staining revealed expression of exogenous neuroligin-1 throughout the dendritic tree of cells at this stage. However, dendritic spines were not detected in the majority of neuroligin-1 IRES GFP infected 14-day-old cells (Figure 5A). Likewise, mEPSCs were virtually absent in neuroligin-1 overexpressing cells at 14 days (4/10 cells with no events, 36 events in 101 minutes from 10 NLG-1 cells; p>0.2 for having one or more detected events, Fisher's exact test, two-tailed; Figure 5B, C). As mEPSCs were infrequent in both groups, we omitted TTX from the bath to include action-potential dependent events (sEPSCs) and increase the number of events. However, we still found a similar lack of excitatory synaptic activity in each condition (control: 5/9 cells with no events, 11 events in 86 minutes of recording from the 9 cells; NLG-1: 4/8 cells with no events, 23 events total in 67 minutes/8 cells; p>0.2 vs. control for frequency (t-test), Fisher's exact test p>0.9 for no events). Given the extremely low number of events in each group/condition, we cannot exclude a small increase in excitatory innervation in the NLG-1 group; however, we conclude that neuroligin-1 overexpression does not cause a substantial increase in excitatory synaptic innervation at this stage.

Although the effects of neuroligin-1 overexpression were selective for excitatory synaptic function at 21 days, it was possible that the lack of excitatory synapses at 14 days post-mitosis might result in a redistribution of exogenous neuroligin-1 to GABAergic synapses. However, overexpression of neuroligin-1 also did not alter the amplitude or frequency of GABAergic mIPSCs (Figure 5D), nor the mIPSC kinetics (Figure 5E; mIPSC 10–90% rise time: control  = 2.1±0.2 ms, NLG-1 = 1.9±0.1 ms; mIPSC half-width: control 17.2 ±1.2 ms, NLG-1 18.5±0.8 ms, n = 14, 9 cells). Thus, neuroligin-1 overexpression did not accelerate the onset of excitatory innervation and did not alter normal GABAergic innervation.

### Neuroligin-1 overexpression transiently increased dendritic process formation

**Figure 6:**
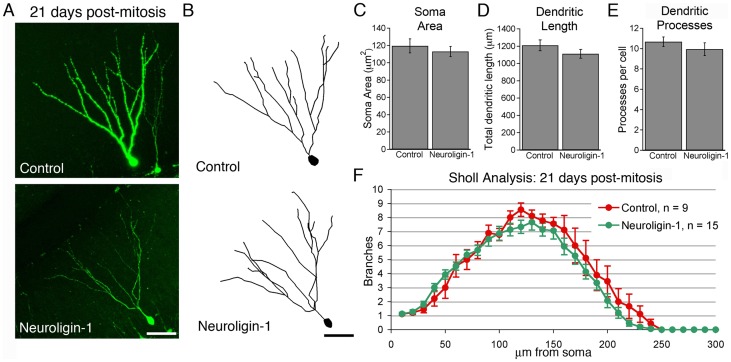
Neuroligin-1 overexpression does not change granule cell soma or dendrite morphology at 21 days post-mitosis. A. Low-power images of 21-day-old granule cells labeled with retroviruses encoding GFP only (above) or neuroligin-1 IRES GFP (below). Scale bar: 50 µm. B. Tracings of the same 21-day-old neurons, used for quantification. Scale bar: 50 µm. C, D, E. Summary data for 21 day old neurons showing no difference in cell soma size, total dendritic length, or dendritic process number between conditions (shown ± SEM; control, n = 9 cells; NLG-1, n = 15 cells; n.s. all conditions). F. Dendritic branch (Sholl) analysis demonstrates a similar overall branching pattern in control and neuroligin-1 overexpressing 21-day-old cells.

During the first weeks post-mitosis, newborn granule cells extend dendrites through the dentate molecular layer [12], establishing the characteristic branching pattern of mature granule cells. As neuroligin-1 overexpression alters neuronal arborization during *Xenopus* tadpole development [45], we examined the dendritic morphology of neuroligin-1 overexpressing cells in the adult mouse. As expected, newborn control granule cell dendrites branched and extended across the entire width of the dentate molecular layer by 21 days post-mitosis (Figure 6A, B). Age-matched neuroligin-1 overexpressing granule cells did not differ significantly from control cells in total dendritic length, number of dendritic processes, or soma cross-sectional area (Figure 6A–E). The Sholl analysis of the dendritic branching patterns for the two groups also did not differ (Figure 6F; p>0.2 for effect of group, two-way repeated measures ANOVA).

**Figure 7:**
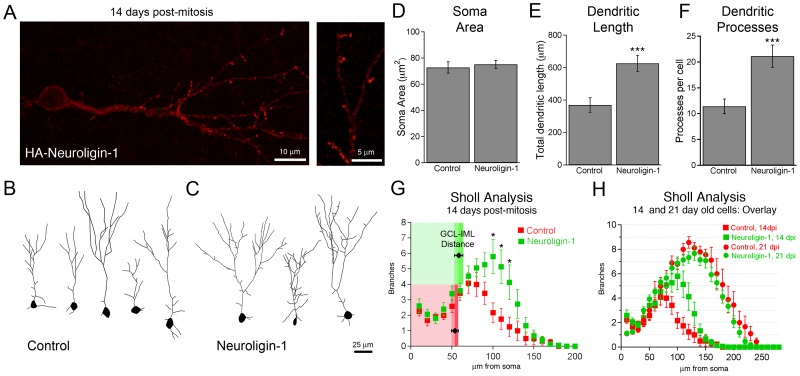
Neuroligin-1 overexpression transiently increases dendritic process number. A. HA-neuroligin-1 expression pattern in 14-day-old adult-born granule cells, visualized with anti-HA staining. Exogenous neuroligin-1 is expressed throughout the dendritic tree (left; scale bar, 10 µm.) and is concentrated along filopodium-like protrusions from the dendritic shaft (right; scale bar: 5 µm). B, C. Dendritic tree morphology of representative 14-day-old neurons shows an increased number of processes along the dendritic tree of neuroligin-1 overexpressing cells. Scale bar: 25 µm. D, E, F. Summary data for 14 day old neurons shows no difference in cell soma size, but a neuroligin-1 mediated increase in total dendritic length and dendritic process number (shown ± SEM, control, n = 12 cells; NLG-1, n = 11 cells; *** p<0.005). G. Dendritic branch (Sholl) analysis demonstrates increased branching along more distal dendrites in neuroligin-1 overexpressing 14-day-old cells (* p<0. 01, two way repeated measures ANOVA). The shaded areas correspond to the average distance from each soma to the granule cell layer (GCL)-inner molecular layer (IML) border in the two groups (n.s.). H. Overlay of Sholl analyses from 14 and 21 day old neurons. Between 14 and 21 days, granule cell dendrites grow to span the full width of the dentate molecular layer and basal dendrites (10–20 µm from soma) retract.

We also performed a morphologic analysis at 14 days post-mitosis, when the dendritic branches are actively expanding. At this stage, neuroligin-1 overexpression increased total dendritic length and the number of dendritic processes, without altering cell soma size (Figure 7B–F). Sholl analysis demonstrated that the increase in branch numbers occurred primarily in more distal parts of the apical dendrite, beginning approximately 80 microns from the soma (Figure 7G; p<0.01 for 100, 110, and 120 micron rings, two-way repeated measures ANOVA with Bonferroni post-hoc test). As the distance from the soma to the inner border of the inner molecular layer (IML) was approximately 60 microns (control  = 54±4 µm, NLG-1 = 58±5 µm, n = 12, 11 cells respectively, p = 0.5; Figure 7G), the increased branching occurred in the inner molecular layer, which contains excitatory afferent fibers that have yet to make functional synapses onto newborn neurons.

Although overlay of Sholl curves from 14 and 21 days (Figure 7H) might seem to indicate that neuroligin-1 overexpression accelerated overall dendritic growth, closer analysis presented a more nuanced picture. First, the number of dendritic processes in neuroligin-1 overexpressing cells actually decreased between 14 and 21 days (NLG-1 at 14 dpi  = 21.1±2.2 processes/cell, NLG-1 at 21 dpi  = 9.9±0.6 processes/cell, n = 15, 15 cells, p<0.001), suggesting that some of these processes regressed as the cells matured. Additionally, the distribution of individual dendritic segment lengths (measured to the closest branch point) demonstrated a preferential increase in very short processes (processes less than 10 µm: control  = 2.8±0.5 processes/cell, NLG-1 = 6.8±1.2 processes/ cell, p<0.01), in contrast with processes between 10–20 µm long (control  = 2.5±0.3 processes/cell, NLG-1 = 2.3±0.5 processes/cell, p = 0.7). Thus the change in cell morphology induced by neuroligin-1 overexpression results in part from an increased number of short filopodium-like processes within the inner molecular layer that subsequently regress. An analysis of exogenous neuroligin-1 localization sheds further light on this observation. Unlike the localization of HA-neuroligin-1 to spine tips at 21 days, exogenous HA-tagged neuroligin-1 was localized more diffusely throughout the dendritic shaft at 14 days, and in highly concentrated stretches along these short dendritic processes (Figure 7A). The association of neuroligin-1 with these short processes suggests a possible role for neuroligin-1 in the formation or stabilization of these processes.

## Discussion

We used retroviruses to overexpress neuroligin-1 in newborn granule cells in adult mice. With this approach, we were able to examine synapse formation *in vivo,* in a context that preserved normal presynaptic structures and activity patterns, and at specific timepoints relative to the onset of excitatory and inhibitory synaptogenesis. Neuroligin-1 properly targeted to dendritic spines, suggesting that the level of expression in our experiments did not alter proper trafficking of exogenous protein. Although neuroligin-1 selectively increased the number of excitatory synapses onto newborn granule cells, it did not accelerate the time of appearance of excitatory synapses, nor have any effects on inhibitory synaptic function.

### Neuroligin-1 and synaptogenesis

At 14-days post-mitosis, newborn granule cells express functional glutamate receptors, and the dendrites of these cells reach across the inner molecular layer [36], [46], where they are surrounded, but apparently not contacted, by glutamatergic nerve terminals [47]. Given that neuroligin-1 can induce ‘artificial’ excitatory synapses onto Human Embryonic Kidney (HEK) cells when co-expressed with NMDA receptors [22], it was surprising that neuroligin-1 did not drive precocious excitatory synaptic development in neurons *in vivo*. Although it is possible that young granule cells lack other crucial components of the post-synaptic apparatus, it seems likely that newborn neurons have a more robust complement of synaptic proteins than a non-neural cell line. Alternatively, excitatory synapse formation might be actively inhibited in young granule cells by some unknown regulatory mechanism, which becomes less active as granule cells mature, such as histone de-acetylation [48]. However, the induction of synapse formation in heterologous cells could also be the result of high levels of overexpression, given that short term transfection approaches *in vitro* generally result in higher levels of expression than are typical for viral-mediated gene transfer. Although we did not precisely quantify the levels of overexpression in our experiments, the relatively sparse label of neurons, particularly in double infection experiments where few cells are co-labeled despite simultaneous injection of a mixed viral suspension, suggests that labeled neurons are typically infected with only a single viral particle. Overall, our results suggest that increased neuroligin-1 expression is not sufficient to accelerate excitatory synapse formation in 14-day-old newborn neurons *in vivo*.

The distinct extracellular milieu of adult animals, such as differences in neurexin isoform expression [49] and the nearby presence of fully mature synapses [50], may fundamentally alter synaptogenesis in adult-born neurons, such that processes critical in embryonic synapse development might not necessarily apply to adult-born neurons. Alternatively, other known synaptogenic molecules, such as the LRRTMs and SynCAM [51], [52], [53], may also play an instructive role in synapse formation onto adult-born neurons. These possibilities highlight the importance of studying the process of synapse formation during adult neurogenesis *in vivo*, as it will help to resolve differences that may result both from technical (*in vitro* vs. *in vivo*) and biological (embryonic vs. adult-born neuron) factors.

### Synapse-specific action of neuroligin-1

Neuroligin-1 is primarily localized at excitatory synapses *in vivo*, and is hypothesized to preferentially play a role in excitatory synapse development [29], [30], [31], [54], [55], whereas neuroligin-2 appears to be primarily involved in inhibitory synapse formation [27], [28], [29], [54], [56], [57], [58]. Despite this apparent synapse-specificity, neuroligin-1 overexpression *in vitro* increases inhibitory synapse formation [27], [28]. In our *in vivo* experiments, the effect of overexpressed neuroligin-1 was specific to excitatory synapses in older cells, and neuroligin-1 did not alter inhibitory synapse number during an earlier period when only GABAergic synapses are present on newborn neurons. Likewise, pan-developmental neuroligin-1 overexpression with an *in vivo* transgenic approach did not affect inhibitory synapses [31]. Given the ability of exogenous neuroligin proteins to form dimers with endogenous proteins [25], high overexpression levels following *in vitro* transfections might alter patterns of neuroligin isoform localization, which could possibly explain the discrepancies between methodologies. Alternatively, *in vitro* models might have fundamentally altered neuroligin trafficking pathways, which are more likely to contribute to mis-localized targeting regardless of expression level.

During the phase of excitatory synaptogenesis in newborn granule cells (2–3 weeks post-mitosis), neuroligin-1 overexpression increased the frequency of mEPSCs, without increasing quantal size, similar to what has been reported in prior studies [25], [26], [59]. Although there are links between neuroligins and presynaptic mechanisms [25], [38], [60], the lack of a change in PPF in our experiments argues against a presynaptic mechanism for the increase in mEPSCs. This result suggests that the primary effect of neuroligin-1 was to increase the number, rather than the strength, of individual synapses. In contrast, overexpression of another postsynaptic molecule, PSD-95 [26], [61] increases mEPSC amplitude, presumably as a result of synaptic AMPA receptor recruitment. Neuroligin-1 overexpressing cells did show increased amplitude and frequency of spontaneous EPSCs, indicating that that action-potential dependent release onto neuroligin-1 overexpressing cells involved the simultaneous release of multiple quanta, presumably at different synapses. The simplest interpretation of this result is that individual axons contact an increased number of dendritic spines similar to the increase in “connection multiplicity” noted during development in hippocampal region CA1 [41].

The adult neuroligin-1 knockout mouse has a reduced NMDAR/AMPAR ratio at mature excitatory synapses, suggesting a role for neuroligin-1 in the retention of NMDARs at synapses [29], [62], [63]. However, the reduced synaptic NMDAR localization seen in mature neuroligin-1 knockout animals is absent in early postnatal (P14–18) knockout hippocampus, suggesting differences in receptor trafficking mechanisms between young and old neurons [53]. Consistent with a possible developmental change in trafficking mechanisms, studies in young organotypic cultures have shown that neuroligin-1 overexpression leads to an enhancement of both AMPAR and NMDAR responses with a slightly greater relative increase in the AMPAR-mediated responses [25], [38]. Our current results are consistent with the results seen in younger neurons (*in vivo* and *in vitro*), further suggesting that *in vivo* differentiation of adult-born neurons may recapitulate aspects of early post-natal development of embryonically produced neurons, as been suggested previously [12], [16], [43].

### Neuroligin-1 overexpression and filopodium-like process formation

Although neuroligin-1 has long been considered a critical player in functional synapse formation, neuroligin-1 overexpression during development alters the dynamics of dendritic process outgrowth in *Xenopus* tadpole tectum [45]. In that study, neuroligin-1 caused dendritic arbors to become more complex and compact, primarily through an increase in filopodilal density and altered filopodial dynamics. The preferential increase in short processes (< 10µm) by neuroligin-1 overexpression in our experiments is consistent with the induction or stabilization of filopodia (as defined by Chen et al.). Live-cell imaging in mouse hippocampal cultures (8–9 div) indicates that neuroligin-1 overexpression can alter filopodial dynamics [64], which might reflect an early stage in synapse formation [65]. The presence of these processes in the inner molecular layer near excitatory afferents, as well as the decrease in process number in neuroligin-1 overexpressing granule cells by 21 days post-mitosis, hints that these processes might represent the very earliest onset of excitatory synapse formation. The lack of filopodium-like processes at 21 days post-mitosis either reflects the selective localization (and function) of neuroligin-1 at synaptic sites, or the transition of the granule cell to a mode of less active filopodium formation.

### Implications for cell-based therapies

Adult-generated newborn granule cells may have a role in recovery from neuronal injury [9], [66], [67], [68], [69], [70], [71], [72], [73], [74], and attempts to engraft neuronal stem cells into the brain for therapeutic purposes is increasingly becoming a reality [75]. However, survival of transplanted cells may not be the main limiting factor in cell-based therapies in the brain, but rather it may be the appropriate functional integration into neural circuits. For example, stem cells engrafted to treat Parkinson’s disease often do not re-establish the normal striatal circuitry, and have been associated with graft-induced dyskinesias [76], [77], [78]. Similarly, stem cell transplantation strategies being investigated in traumatic brain injury show that transplanted cells integrate poorly, if at all [79]. Our results suggest that newborn neurons could provide a valuable experimental template to examine molecules controlling the successful integration of new neurons into existing circuits.
